# Portovenography Findings Following Partial Polypropylene Versus Thin Film Band Attenuation of a Single Congenital Extrahepatic Portosystemic Shunt: A Prospective Randomized Study in Dogs

**DOI:** 10.3390/vetsci10050353

**Published:** 2023-05-15

**Authors:** Victoria Lipscomb, Mickey Tivers, Anne Kummeling, Freek van Sluijs

**Affiliations:** 1 Department of Clinical Sciences and Services, Royal Veterinary College, University of London, Hatfield, Hertfordshire AL9 7TA, UK; 2 Paragon Veterinary Referrals, Wakefield, West Yorkshire WF1 2DF, UK; mickey.tivers@paragonreferrals.co.uk; 3 Department Clinical Sciences, Faculty of Veterinary Medicine, 3584 CL Utrecht, The Netherlands; a.kummeling@uu.nl; 4 Department for Small Animals, Vetsuisse Faculty, CH-8057 Zurich, Switzerland; f.j.vansluijs@uu.nl

**Keywords:** mesenteric portovenography, congenital vessel anomaly, multiple acquired shunts, residual shunt flow, congenital

## Abstract

**Simple Summary:**

Dogs may be born with an abnormal vessel that bypasses their liver (congenital portosystemic shunt), causing a variety of clinical signs due to toxins building up in the bloodstream. Closing the abnormal vessel completely in a one-stage surgery is ideal but cannot always be tolerated, in which case partial closure with a material that induces ongoing shunt narrowing by inflammation may be chosen. Some dogs receive a second surgery to achieve full closure of the abnormal vessel if ongoing inflammation around the shunt has not managed to produce full shunt closure. There are inaccuracies reported with trying to assess if the abnormal vessel has progressed to full closure using regular ultrasound and computed tomography (CT) imaging to follow up after surgery. The objective of this study was to compare intra-operative X-ray findings (portovenogram) using a dye (contrast) injected directly into the abnormal vessel at a routine second surgery to accurately determine whether different methods of narrowing the shunt achieved full shunt closure over time. Twenty-four dogs were enrolled, 12 received partial polypropylene suture ligation, and 12 received partial thin film band shunt attenuation. Intra-operative portovenography three months after the first surgery demonstrated that nine dogs (75%) that had their shunt narrowed with a thin film band had achieved complete shunt closure versus two dogs (16.7%) that had their shunt narrowed with polypropylene suture. Multiple acquired shunts, which are not congenital but develop in response to excessive pressure within the liver’s circulation (due to narrowing or closure of the single congenital shunt), developed in two dogs receiving thin film band attenuation of their congenital shunt.

**Abstract:**

The objective was to conduct a prospective, randomized study to compare mesenteric portovenogram findings following partial polypropylene suture versus thin film band extrahepatic portosystemic shunt attenuation in dogs. Dogs with extrahepatic portosystemic shunts that could not tolerate complete acute shunt closure received a partial attenuation with either a polypropylene suture or synthetic polymer thin film band. At a routine second surgery three months after shunt patency, missed shunt branches and/or development of multiple acquired shunts were assessed using intra-operative mesenteric portovenography. Twenty-four dogs were enrolled, 12 received partial polypropylene suture ligation, and 12 received partial thin film band shunt attenuation. Intra-operative mesenteric portovenography three months later demonstrated that nine dogs (75%) in the thin film band group had achieved complete shunt closure versus two dogs (16.7%) in the polypropylene suture group, which was significantly different (*p* = 0.004). No dogs in the polypropylene suture group and two dogs (16.7%) in the thin film band group developed multiple acquired shunts. This is the first study directly comparing follow-up intra-operative mesenteric portovenography imaging findings between two methods of partial portosystemic shunt attenuation in dogs. The study provides accurate information on the rates of complete anatomical shunt closure and development of multiple acquired shunts following partial shunt attenuation with either synthetic polymer thin film band or polypropylene suture.

## 1. Introduction

Surgical treatment of a congenital portosystemic shunt (CPSS) aims to restore portal hepatic perfusion by CPSS closure but may lead to life-threatening portal hypertension if the portal vein and its branches are underdeveloped, which may apply to as many as 84% of dogs [1]. Thin film banding (TFB), ameroid ring constrictor (ARC), or polyacrylic acid-silicone devices aim to induce the gradual and eventual complete occlusion of the shunt, while partial suture ligation leaves the shunt partially open [2,3,4,5]. Continued shunting after surgery may occur because of residual flow through the shunt due to incomplete occlusion, a missed branch due to technical error at surgery, and/or the development of multiple acquired shunts (MAS) [6,7,8,9,10].

There is more information in the literature describing peri-operative survival and short and long-term clinical outcome of dogs undergoing portosystemic shunt TFB attenuation (assessed by retrospective review of medical records, owner questionnaires, clinician examination, and/or biochemical testing) than post-operative imaging to assess anatomical shunt closure, missed branches, and/or acquired portosystemic shunt development following TFB attenuation. A systematic review and meta-analysis determined that there were eleven studies with a total of 269 dogs evaluating TFB extrahepatic shunt attenuation in dogs [11]: clinical outcome was available for 139 dogs, of which 136 dogs (97.8%) had a successful clinical outcome which was defined as either no clinical signs/no medical treatment or minimal/no clinical signs on medical treatment [2,10,11,12,13,14,15]. Follow-up surgical visualization or imaging outcome was available for 90 dogs, with 51 (56.7%) dogs having a closed shunt without MAS [9,10,11,12,15,16,17].

Reasons for post-operative shunt imaging not being performed include cost and availability of advanced imaging (scintigraphy, portovenography, computed tomography (CT) angiography, magnetic resonance angiography), the need for sedation or anesthesia to perform the imaging, and the invasiveness of portovenography techniques. Nevertheless, a systematic review and meta-analysis of the treatment of congenital extrahepatic portosystemic shunts in dogs concluded that further blinded, randomized studies comparing different treatment modalities, which routinely include postoperative imaging to assess shunt closure and acquired portosystemic shunt development, are essential [11]. Differentiating between MAS, residual flow through the original shunt, or missed shunt branches is critical for further patient management decisions as the treatment options for the residual flow or missed shunt branches may include additional surgery to correct the problem, whereas MAS cannot be treated surgically [8,18].

During the transition at our institution from routinely performing two surgeries in dogs that only tolerated partial suture ligation at the first surgery (with the aim of complete shunt ligation at a second surgery three months later) to placing a thin film band to partially attenuate an extrahepatic shunt, we had an opportunity to prospectively enroll dogs routinely expected to receive two shunt surgeries into a study where follow up intra-operative imaging was performed in all cases at the second surgery. In this unique population of dogs, the application of either polypropylene suture or thin film band to partially attenuate the shunt could be randomized, and the effect on shunt closure assessed during follow-up intra-operative mesenteric portovenography at the second surgery.

The aim of this study was to conduct a prospective randomized trial to compare mesenteric portovenogram findings following partial polypropylene (P) suture versus thin film band (TFB) partial shunt attenuation. In one previously reported study, 10 of 16 dogs (63%) of the shunts treated with TFB had a normal shunt fraction on follow-up portal scintigraphy 10 weeks after surgery, and of those that were still shunting, mesenteric portovenography demonstrated half (3 dogs, 18.6%) were due to ongoing shunting through the original shunt and half (3 dogs, 18.6%) were due to MAS [15]. Another study utilizing a synthetic polyethylene TFB reported persistent shunting in 17% of dogs [19]. A study utilizing partial polypropylene attenuation reported that all 17 dogs that underwent a second surgery demonstrated ongoing shunting through the original shunt on mesenteric portovenography [20]. Therefore, our hypothesis was that partial attenuation of the shunt with TFB would result in a higher number of completely closed shunts than partial attenuation with P.

Multiple acquired shunts may develop in up to 20% of animals undergoing surgical treatment for a single congenital portosystemic shunt, occurring when the intrahepatic portal capacity is exceeded [2,4,15]. However, the factors that may contribute to MAS development in a proportion of dogs following surgical treatment of their shunt are unknown. We were also interested in this study to assess the occurrence of multiple acquired shunts in both groups.

## 2. Materials and Methods

The study was designed as a prospective, randomized clinical trial for dogs undergoing partial shunt attenuation of a single congenital extrahepatic portosystemic shunt between January 2012 and December 2016. Ethical approval was confirmed by the primary author’s institution’s ethical board prior to the start of this study, and all owners gave prospective written consent for their dog’s participation in the study (ref 2011–1099).

**Figure 1:**
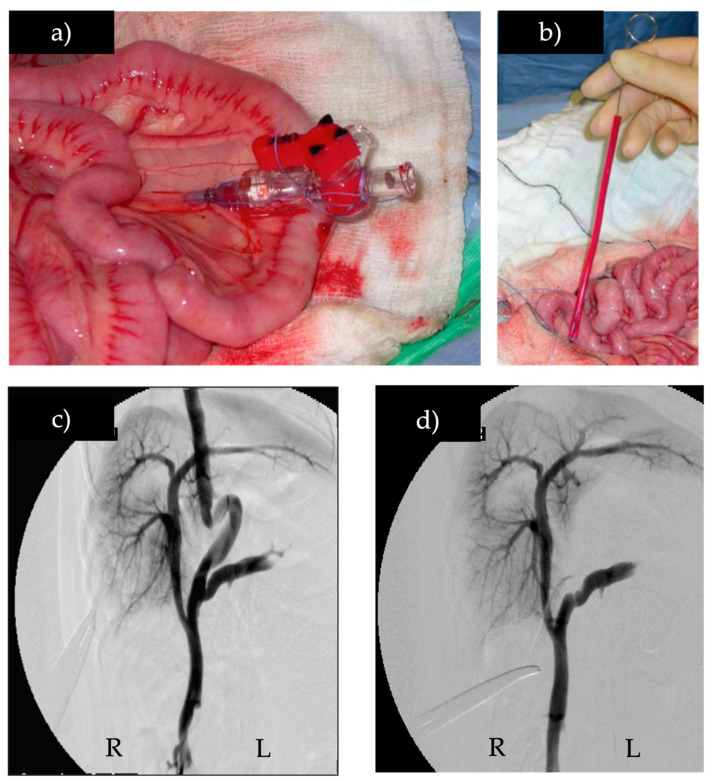
Intra-operative mesenteric portovenography before and after temporary full shunt occlusion, (**a**) catheter in the mesenteric vein, (**b**) Rummel tourniquet being placed to temporarily occlude shunt, (**c**) intra-operative mesenteric portovenogram of a portoazygous shunt, (**d**) intra-operative mesenteric portovenogram of the same portoazygous shunt during temporary full occlusion with the Rummel tourniquet. (R) right, (L) left.

Treatment was similar in all dogs except for the material that was used to partially attenuate the shunt. Shunts were temporarily completely occluded intra-operatively (Figure 1), and the presence of portal hypertension was assessed. Changes in mean arterial pressure, central venous pressure, heart rate, and CO2ET ≥ 20% from starting values and intestinal or pancreas cyanosis were considered evidence of portal hypertension. Dogs in which portal hypertension did not develop during full shunt occlusion during surgery had their shunts completely closed with sutures and were excluded from the study. Dogs in which portal hypertension developed were randomly assigned using freely available online randomizing software (Research Randomizer v4.0 https://www.randomizer.org, accessed 25^th^ August 2011) to one of two interventions: partial attenuation with an autoclaved double folded four mm wide, clear, non-proprietary, synthetic polymer TFB with a uniform thickness (mean 55.3 μm, SD 18.33) or with two metric polypropylene (P) sutures (Prolene, Ethicon). Intra-operative mesenteric portovenography was used to assess the optimal placement of P or TFB around a single congenital extrahepatic shunt vessel close to the shunt insertion in the systemic circulation, proximal to any contributing shunt branches (Figure 1). Digital subtraction intra-operative mesenteric portovenography was performed using a mobile C-arm fluoroscope (Siremobil, Siemens). A mesenteric vein was catheterized with the largest bore catheter possible (21 or 23 gauge), and a bolus of 1 mL/kg of iodinated contrast medium (Omnipaque 300, Amersham Health) was injected as quickly as possible into the mesenteric vein for each portovenogram. Images were acquired at a rate of 1/s from the start of contrast medium injection until hepatic portal opacification faded. The degree to which the shunt could be narrowed without inducing portal hypertension was assessed by gauged ligation with P, as previously described [21]. In the P group, the suture was left in place when the maximum degree of narrowing was obtained. In the TFB group, the suture was removed, and the TFB was tightened to the same diameter as the ligature that was removed, using at least two metal clips. In both groups, another loose polypropylene suture was left in situ around the shunt so that if the shunt was still patent with no MAS at the second surgery, the shunt could be fully closed using the preplaced suture, provided that this did not result in portal hypertension. Intra-operative portovenography findings at the second surgery, three months after the first surgery, were analyzed for the presence or absence of residual flow through the original shunt, the presence or absence of missed shunt branches, and the presence or absence of MAS.

Continuous data were assessed for normality graphically. Non-parametric data were reported as median and range. Differences in age, body weight, and the time interval between the first and second surgery between the two groups were analyzed with a Mann–Whitney U Test. We estimated that approximately 70% of the shunts treated with TFB would close completely based on published studies [8,15,19] and that none of the shunts treated with P would close completely [20]. Based on these assumptions, at least 16 cases (eight per group) were required to find a statistically significant difference, assuming a beta of 0.8 and an alpha of 0.05. Differences in the presence or absence of residual shunt flow between the two groups were analyzed with a Fisher’s Exact Test. Differences were considered statistically significant at *p* < 0.05. All calculations were performed using a statistical software program (IBM SPSS Statistics Version 28).

## 3. Results

Fifty-four dogs received surgical treatment of a congenital extrahepatic portosystemic shunt, of which 30 dogs received complete acute shunt ligation and were excluded from the study. Twenty-four dogs were prospectively enrolled in the study, 12 received partial P suture ligation, and 12 received partial TFB attenuation (Table 1 and Table 2). There were 11 males, three of whom were neutered. There were 13 females, 10 of whom were neutered. Breeds included three Maltese Terriers, three Pugs, two Bichon Frises, two Labrador Retrievers, two Miniature Schnauzers, two Dachsunds, five crossbreeds, and one each of Golden Retriever, Skye Terrier, Shih Tzu, Chihuahua, and Cockapoo (Table 1 and Table 2). The median age at first surgery was 6 months (range 3 months to 11 months) for P and 8 months (range 4 months to 34 months) for TFB (Table 1 and Table 2). The median body weight at first surgery was 4 kg (range 2 kg to 10.3 kg) for P and 4.4 kg (range 1.5 kg to 19.5 kg) for TFB (Table 1 and Table 2). There was no significant difference in body weight or age between the P and TFB groups (p = 0.932 and p = 0.128, respectively).

Intra-operative mesenteric portovenography at both the first and second surgery confirmed optimal placement of P or TFB around a single congenital extrahepatic shunt with no missed shunt branches in all dogs. Intra-operative portovenography was performed at the second surgery at a median of 3.5 months (range 3 months to 11 months) after the first surgery (Table 3 and Table 4). There was no difference in the time interval between the first and second surgeries between the P and TFB groups (p = 0.101). Intra-operative mesenteric portovenography at the second surgery demonstrated 9 of 12 (75%) dogs in the TFB group had progressed to complete closure of their shunt compared to 2 of 12 (16.7%) dogs in the P group (Table 3 and Table 4). This difference was statistically significant (p = 0.004). Additionally, none of the dogs in the P group and two dogs (16.7%) in the TFB group developed MAS (Table 3 and Table 4). Both dogs in the TFB group that developed MAS still had an original patent shunt alongside the development of MAS (Table 3).

## 4. Discussion

This is the first prospective, randomized study comparing follow-up intra-operative mesenteric portovenography imaging findings between two methods of partial extrahepatic portosystemic shunt attenuation in dogs. Post-operative imaging has been recommended as essential to assess shunt closure and the development of multiple acquired shunts to complement other follow-up clinical information when comparing different methods of congenital portosystemic shunt attenuation in dogs [11,22]. In particular, differentiation of MAS from residual flow through the original shunt or missed shunt branches is critical as the latter two scenarios both have the potential to be further corrected surgically, whereas MAS can only be managed medically [8,18].

The results of this study demonstrate that approximately 75% of dogs not able to tolerate complete acute shunt ligation and receiving synthetic polymer thin film band partial shunt attenuation at our institution are expected to achieve complete shunt closure over time. A second surgery is no longer routinely performed at our institution following partial TFB shunt attenuation, but an additional loose polypropylene suture is always left around the shunt in case it may be needed at a future surgery due to recurrence or persistence of clinical signs. The high rate of thin film band complete shunt closure over time was anticipated in our original hypothesis as it is suggested that various sources of thin film band elicit an inflammatory reaction that promotes ongoing vascular attenuation [23], whereas polypropylene is considered an inert material without this property. It is interesting to note that a small number of shunts partially ligated with polypropylene suture in this study also demonstrated complete shunt closure over time; the mechanism for this is unknown.

The 75% complete shunt closure over time following partial shunt attenuation with TFB demonstrated in this study is similar to the results of a previous study which reported 15 of 49 (31%) dogs that received partial TFB attenuation of their shunt had persistent shunting identified by diagnostic imaging (CT [n = 7], ultrasound [n = 6)], and portovenography [n = 2]) at a median of 5 months (range, 2–54) postoperatively) [8]. In another study where 16 dogs received partial shunt attenuation with a thin film band, three dogs (18.6%) were identified with residual flow through the original shunt, and three dogs (18.6%) developed multiple acquired shunts by 10 weeks post-operatively using scintigraphy and mesenteric portvenography [15]. In another study, persistent shunting was identified in nine of the 53 (17%) dogs at short-term follow-up, at a median of 180 days (range 85 to 1460) post-operatively [19]. Four dogs were diagnosed by ultrasound, three by CT, one by both ultrasound and CT, and one by exploratory celiotomy [19]. The causes of persistent shunting were previously unidentified branches or second shunts in three dogs and persistent flow through the attenuated vessel in two dogs; none had MAS [19]. In another study where five dogs had a TFB placed, and 15 dogs had an ameroid constrictor placed, transplenic portal scintigraphy 3 months later demonstrated that 14 dogs had a closed shunt, three developed MAS, and two dogs had persistent shunting through the original shunt (the scintigraphy outcome was unclear in the remaining dog) [22]. There is one study that reports quite a different shunt closure rate from ours and other studies, with only 29% full shunt closure following TFB shunt attenuation compared to 71% full shunt closure in the same study for dogs that had an ameroid constrictor placed [24]. The use of various sources of non-proprietary thin film bands introduces variability when comparing results between institutions, which may be one reason for the differences in outcome seen between institutions, although it is interesting to note that rates of complete shunt closure over time, assessed via imaging, for different sources of thin film band are often broadly similar. The rate of TFB progression to complete closure following initial partial constriction of the shunt at the surgery in this study, assessed by mesenteric portovenography, is also broadly similar to the results of a prospective study of 20 dogs undergoing thin film band attenuation of a shunt without initial shunt constriction which revealed complete shunt occlusion on post-operative CT angiography in 13 dogs (65%) [9]. It is very interesting to note that the results of our study, where the selection criteria only included dogs that were not able to tolerate complete acute shunt ligation, were broadly similar to other studies where this selection criterion was not applied, i.e., TFB partial attenuation was performed on the entire population of dogs presenting for surgical treatment of an extrahepatic shunt.

A study of 168 dogs with an ameroid constrictor placed (without intra-operative imaging) on their extrahepatic shunt achieved an excellent long-term outcome in 80% of dogs despite 21% persistent shunting identified in those that underwent portal scintigraphy 6-10 weeks after surgery [4]. The absence of continued shunting was one of the factors predictive of an excellent outcome. However, it remains unclear what the clinical significance of a small degree of postoperative residual shunt flow may be [4]. Whilst therefore not essential, as evidenced by numerous studies reporting excellent outcomes using a variety of techniques that do not utilize intra-operative imaging [2,3,4,5,10,12,14,19,22,25,26,27,28,29], one of the benefits of intra-operative mesenteric portovenography if available, is that additional shunt branches or second shunts are unlikely to be missed ensuring optimal shunt attenuation location. Although uncommon, there are reports of this occurring: in one study, postoperative CT angiography demonstrated that the thin film band was in a suboptimal location in eight dogs (40%) [9]; in another study, CT angiography showed a missed shunt branch cranial to suboptimal ameroid ring constrictor location in two dogs (9%) [7], and in another study, a previously missed shunt branch or second shunt was identified at follow-up imaging in three dogs [19]. A study comparing pre-operative CT angiography and intra-operative mesenteric portovenography suggested that CT angiography does not replace intra-operative mesenteric portovenography after temporary full ligation, which confirms a single shunt has been occluded, that there are no missed branches, and provides information on intrahepatic portal vascularity [30].

Given the information available, the best imaging technique for assessing postoperative residual shunt flow, missed shunt branches, and/or development of MAS is not clear. One study compared CT angiography versus splenoportography three months after thin film band shunt attenuation in 20 dogs, revealing a substantial to perfect agreement between three independent reviewers for splenoportography and a slight to a moderate agreement for CT angiography [17]. More CT angiography studies were classified as still shunting at follow-up compared to splenoportography, and a significant correlation between CT angiography and splenoportography for identification of residual shunting was present in only one of three observers [17]. Portography is more selective and dynamic, which is likely to increase its reliability for assessing residual shunt flow. An issue contributing to the poor inter-observer agreement for CT angiography was suggested to be the difficulty discriminating between inflammatory reaction and residual shunt flow [17]. Further issues with post-operative CT angiography may include the presence of a metal clip or metal ameroid constrictor artifact at the level of shunt occlusion, which may interfere with imaging interpretation of residual shunt flow [7,31]. In a study of 22 dogs utilizing CT angiography immediately before and at least eight weeks after the placement of ameroid ring constrictors, residual shunt flow was present in four dogs (18%), MAS developed in two dogs (9%), and a missed shunt branch cranial to the suboptimal ameroid ring constrictor location was demonstrated in two dogs (9%) [7]. A plastic ameroid ring constrictor was placed in 17 of the dogs in that study to reduce artifacts on the CT angiogram [7]. A retrospective multicenter study of 206 dogs undergoing ameroid constrictor placement for an extrahepatic shunt demonstrated 24% persistent shunting via post-operative nuclear scintigraphy [32]. However, scintigraphy cannot differentiate between residual flow, missed shunt branches, and/or MAS as the reason for persistent shunting. Abdominal ultrasound has also been used for post-operative imaging follow-up; a retrospective, multi-institutional study of 23 dogs that received an ameroid constrictor and 26 dogs that received thin film band (with no initial constriction of the shunt) for attenuation of a single extrahepatic portosystemic shunt suggested that residual shunting was suspected to be higher in dogs treated with thin film band (32%) versus an ameroid constrictor [10]. No MAS were reported, and the findings of residual shunting in the TFB dogs documented by ultrasonography were corroborated in five dogs that also had follow-up CT angiography [10]. Ultrasonography may be a reliable (as well as readily available and relatively cheap) method of assessing residual shunt flow versus MAS [18], although it is highly dependent on the skill and training of the operator.

Multiple acquired shunts may develop in up to 20% of animals undergoing surgical treatment for a single congenital portosystemic shunt, occurring when the intrahepatic portal capacity is exceeded [2,4,15], although factors contributing to this development in a proportion of dogs are unknown. The 0–16.7% rate of MAS demonstrated in our study is similar to other reports, which is again interesting given the selection criteria for this study which excluded dogs that could tolerate complete acute shunt closure. A study of follow-up mesenteric portovenography in 10 dogs with elevated serum bile acid concentrations following partial shunt attenuation with silk reported that six dogs had residual flow through their shunt and four dogs had MAS [6]. The high rate of MAS in that study is likely due to the population selected [6]. Whilst one might speculate that MAS is more likely following the application of a material that is expected to induce ongoing shunt closure over time (both the dogs with MAS in our study had TFB applied), it is also interesting to note that in our study both these dogs still had an original patent shunt alongside the MAS which suggests that intrahepatic portal capacity can still be exceeded despite an ongoing route for blood flow through a still patent shunt. The reason(s) why some dogs but not others develop MAS following surgical treatment of their shunt is still very unclear.

Limitations of this study include the fact that multiple surgeons and anesthetists participated, albeit following the prospective study design and standard patient management protocol. The sample size was estimated to be adequate to assess the main hypothesis based on a power calculation prior to commencing the study. However, greater group sizes would have been useful, albeit not practical, given the unique population of study dogs that were enrolled prior to evolving and changing the protocol at our institution. Intra-operative mesenteric portography at a second surgery was the standard protocol in our institution at the time of performing this study, however acquiring the expertise, training, and equipment to perform splenoportography in the future would allow us to continue to benefit from the increased accuracy of portography over ultrasound and CT without the need for another surgery.

## 5. Conclusions

This study represents a unique opportunity to gain prospective, randomized follow-up intra-operative mesenteric portovenography imaging information on shunt closure in dogs following partial polypropylene suture versus partial thin film band attenuation. The results of this study have evolved our practice such that revision shunt surgery, including follow-up intra-operative mesenteric portovenography, is only performed for extrahepatic shunts if clinical signs persist, significant residual shunt flow is identified via other post-operative imaging methods (ultrasonography and/or CT angiography), and it is deemed that repeat exploratory coeliotomy for possible further shunt correction would be beneficial. In the future, large, prospective studies of other post-operative imaging techniques are recommended to accurately assess the rates of complete shunt closure and development of MAS and to determine the most appropriate imaging technique for this purpose following congenital portosystemic shunt attenuation in dogs.

## Figures and Tables

**Table 1:** Randomized dog enrolment number, body weight (kg), age (months), and breed of the 12 dogs that received TFB shunt attenuation.

TFB Attenuation	Body Weight (kg)	Age at First Surgery (Months)	Breed
Dog 1	19.5	8	Labrador Retriever
Dog 2	3.6	4	Miniature Schnauzer
Dog 6	17.8	6	Golden Retriever
Dog 7	6.6	34	Pug
Dog 12	3	18	Maltese Terrier
Dog 15	1.5	10	Chihuahua
Dog 17	2	4	Pug
Dog 19	4.8	18	Bichon Frise
Dog 20	5.3	8	Pug
Dog 21	7.4	20	Crossbreed
Dog 22	3.7	4	Cockapoo
Dog 24	4	6	Shih Tzu

**Table 2:** Randomized dog enrolment number, body weight (kg), age (months), and breed of the 12 dogs that received polypropylene shunt attenuation.

Polypropylene Suture Attenuation	Body Weight (kg)	Age at First Surgery (Months)	Breed
Dog 3	5.5	11	Bichon Frise
Dog 4	3	5	Crossbreed
Dog 5	2.7	3	Miniature Schnauzer
Dog 8	5.4	10	Dachsund
Dog 9	2.1	7	Maltese Terrier
Dog 10	3.7	7	Dachsund
Dog 11	2	4	Maltese Terrier
Dog 13	10.3	5	Skye Terrier
Dog 14	6.1	7	Crossbreed
Dog 16	6.3	4	Crossbreed
Dog 18	4	6	Crossbreed
Dog 23	4	6	Labrador Retriever

**Table 3:** Interval to second surgery (months), whether or not the shunt was found to be occluded at the second surgery (Y = yes, N = no), and whether or not MAS were documented at the second surgery (Y = yes, N = no) in the 12 dogs that received TFB attenuation.

TFB Attenuation	Interval between Surgeries (Months)	Shunt Occluded at 2nd Surgery	MAS at 2nd Surgery
Dog 1	6	N	Y
Dog 2	4	Y	N
Dog 6	3	N	Y
Dog 7	4	N	N
Dog 12	3	Y	N
Dog 15	3	Y	N
Dog 17	5	Y	N
Dog 19	4	Y	N
Dog 20	4	Y	N
Dog 21	11	Y	N
Dog 22	7	Y	N
Dog 24	3	Y	N

**Table 4:** Interval to second surgery (months), whether or not the shunt was found to be occluded at the second surgery (Y = yes, N = no), and whether or not MAS were documented at the second surgery (Y = yes, N = no) in the 12 dogs that received polypropylene attenuation.

Polypropylene Suture Attenuation	Interval between Surgeries (Months)	Shunt Occluded at 2nd Surgery	MAS at 2nd Surgery
Dog 3	3	Y	N
Dog 4	3	N	N
Dog 5	4	N	N
Dog 8	3	N	N
Dog 9	4	N	N
Dog 10	3	N	N
Dog 11	4	N	N
Dog 13	3	N	N
Dog 14	6	N	N
Dog 16	3	Y	N
Dog 18	3	N	N
Dog 23	3	N	N

## Data Availability

The data presented in this study are available on request from the corresponding author. The data are not publicly available due to this being a small non-funded study.
